# Electroacupuncture Improved Chronic Cerebral Hypoperfusion-Induced Anxiety-Like Behavior and Memory Impairments in Spontaneously Hypertensive Rats by Downregulating the ACE/Ang II/AT1R Axis and Upregulating the ACE2/Ang-(1-7)/MasR Axis

**DOI:** 10.1155/2020/9076042

**Published:** 2020-02-26

**Authors:** Peipei Feng, Zemin Wu, Hao Liu, Yafang Shen, Xu Yao, Xinwei Li, Zui Shen

**Affiliations:** ^1^Department of Acupuncture and Moxibustion, Tongde Hospital of Zhejiang Province, Hangzhou, China; ^2^Department of Neurobiology and Acupuncture Research, The Third Clinical Medical College, Zhejiang Chinese Medical University, Key Laboratory of Acupuncture and Neurology of Zhejiang Province, Hangzhou, China

## Abstract

Electroacupuncture (EA) can effectively alleviate anxiety disorders and memory impairments caused by various neurodegenerative diseases; however, the molecular mechanisms underlying its neuroprotective effects are unclear. Previous studies have shown that the renin-angiotensin system (RAS) comprises of two axes with mutual antagonism: the classical angiotensin converting enzyme/angiotensin II/angiotensin II type 1 receptor (ACE/Ang II/AT1R) axis and the protective angiotensin converting enzyme 2/angiotensin-(1-7)/Mas receptor (ACE2/Ang-(1-7)/MasR) axis. In this study, we observed that chronic cerebral hypoperfusion (CCH) mediated anxiety-like behavior and memory impairments in spontaneously hypertensive rats (SHR) via upregulation of the hippocampal classical axis (ACE/Ang II/AT1R) and the partial hippocampal protective axis (ACE2/Ang-(1-7)). However, Ang II levels were much higher than those of Ang-(1–7), indicating that the ACE/Ang II/AT1R axis plays a dominant role in the comorbidity of CCH and hypertension. Moreover, candesartan cilexetil (Canc) and perindopril (Peril) were used as positive control drugs. We found that EA, Canc, and Peril attenuated CCH-induced anxiety-like behavior and memory impairments in SHR, potentially via downregulation of the hippocampal classical axis (ACE/Ang II/AT1R) and upregulation of the whole hippocampal protective axis (ACE2/Ang-(1-7)/MasR). These results suggest that EA therapy for CCH with hypertension may be mediated by two hippocampal RAS axes.

## 1. Introduction

Chronic cerebral hypoperfusion (CCH) is a common pathophysiological state of the central nervous system [1, 2]. Long-term CCH can trigger neurodegeneration and eventually lead to progressive cognitive dysfunction [3–5]. Clinical research has shown that CCH is the common pathological foundation of Alzheimer's disease, vascular dementia, vascular cognitive impairment, and other neurodegenerative diseases [6–8]. Therefore, understanding the mechanisms underlying CCH will facilitate the development of therapeutic strategies for the prevention and treatment of CCH-induced neurodegeneration [6].

The pathological and neuroprotective mechanisms of CCH are complicated. The function and structural integrity of the brain depend on a blood supply consistent with changes in its energy needs; therefore, adequate cerebral blood flow is a key factor in maintaining normal brain function [9]. In humans, CCH is not an isolated pathophysiological phenomenon, but rather occurs alongside other vascular risk factors, including hypertension [10]. Therefore, in order to better replicate the pathophysiology of human diseases, CCH should be studied alongside other vascular risk factors, for example hypertension. In addition, accumulating evidence suggests that cerebral hypoperfusion and hypertension have an interdependent relationship and promote the development of memory impairment [10–13]. CCH [14, 15] and hypertension [16] are risk factors for anxiety, but the effect of their comorbidity on anxiety is unclear. Therefore, the role of CCH with hypertension in the development of anxiety and memory impairment requires elucidation.

The renin-angiotensin system (RAS) comprises of two axes with mutual antagonism: the classical angiotensin converting enzyme/angiotensin II/angiotensin II type 1 receptor (ACE/Ang II/AT1R) axis and the alternative angiotensin converting enzyme 2/angiotensin-(1-7)/Mas receptor (ACE2/Ang-(1-7)/MasR) axis. Studies have confirmed that these two axes in the brain tissue can exert neuronal damage and neuroprotection, respectively. Studies have confirmed that the brain RAS plays an important role in the pathogenesis of CCH [17]. The RAS is also a key system in the regulation of blood pressure [18] and in the process of vascular aging [19]. Furthermore, the over-activation of the RAS protective axis can reduce anxiety-like behavior [20]. Increasing evidence has shown that the brain RAS is related to memory impairments in many neurodegenerative diseases [21]. However, it is unclear how the brain RAS, especially the interaction of the two axes, is involved in CCH with hypertension and the regulation of emotion and memory-related behaviors.

Our previous studies have shown that electroacupuncture (EA) could alleviate cognitive impairment in rats with chronic cerebral ischemia [22] and aged rats [23]. Furthermore, accumulating evidence has demonstrated that EA can effectively alleviate anxiety disorders [24] and memory impairments caused by Alzheimer's disease [25, 26] and vascular dementia [27]. However, whether EA regulates CCH with hypertension and whether this is related to the RAS remain unclear. In this study, we investigated the anxiolytic and memory-ameliorating effects of EA in spontaneously hypertensive rats (SHR) with CCH and the potential role of the brain RAS in the neuroprotective effects of EA.

## 2. Materials and Methods

### 2.1. Animals and Groups

Male SHR (12 weeks old, weighing 250−300 g) were purchased from Vital River Laboratory Animal Technology Co., Ltd., Beijing, China. The rats were housed in groups of four in plastic cages with soft bedding at the University Animal Care Facility under an artificial 12/12 h light-dark cycle. Animals received food and water ad libitum, and a constant room temperature of 23−25°C and relative humidity of 40−70% were maintained. All animal procedures performed in this work were in accordance with the Regulations for the Administration of Affairs Concerning Experimental Animals and were approved by the Animal Care and Welfare Committee of Zhejiang Chinese Medical University, Zhejiang, China.

SHR were randomly divided into sham (sham), CCH model (model), CCH+candesartan cilexetil (Canc), CCH+perindopril (Peril), and CCH+electroacupuncture (EA) groups.

### 2.2. CCH Model

The modified two-vessel occlusion (2VO) method of Cechetti et al. was used to prepare the CCH model [28]. Briefly, after inhalation of isoflurane for anesthesia, a 1 cm incision was made in the middle of the neck of the rat, and the vagus nerve was dissected to expose the common carotid artery. The right common carotid artery was ligated with a 5-0 surgical suture. The wound was sutured layer by layer. The left common carotid artery was ligated in the same manner one week later. The sham group underwent surgery to dissect the vagus nerve without common carotid artery ligation. Strict aseptic conditions were maintained during and after surgery to prevent infection.

### 2.3. EA and Drug Treatment

EA was performed on the rats at the acupoints of Baihui (GV 20) and Dazhui (GV 14) each day during the experimental procedure. Sterilized disposable stainless steel acupuncture needles (Huatuo, Suzhou Medical Co. Ltd., Jiangsu, China) with a 0.35 mm diameter were inserted as deep as 5 mm. The GV 20 acupoint is located above the apex auriculate in the midline of the head. The GV 14 acupoint is located on the posterior midline and in the depression below the spinous process of the 7^th^ cervical vertebra in the prone position. The ends of the needles were attached to a pair of electrodes from an electrical stimulator (HANS 200E, Huawei Co. Ltd., Beijing, China). The EA parameters were set as follows: a constant square wave current output (15 Hz), with the intensities remaining at 1 mA (causing slight vibration of the muscles around the acupoints) for 30 min once a day starting from day 1 postoperatively. In the whole procedure, all rats remained calm without any struggling or vocalizing. Rats in the drug groups received drugs dissolved in physiological saline via intragastric administration at a dose of 1 mg/kg/day for 4 weeks.

### 2.4. Regional Cerebral Blood Flow (rCBF) Monitoring

rCBF was monitored using laser Doppler flowmetry (Probe 403 connected to PeriFlux 5000; Perimed AB, Sweden) following the method of Farkas et al. [29]. After anesthesia was induced using 5% isoflurane, the rats were given a breathing mask and anesthesia was maintained with 2% isoflurane until the end of modeling. The operating table was heated to maintain a body temperature of 37°C. After iodine disinfection followed by alcohol deiodination, a 1 cm long transverse incision was made between the right eye of the rat and the external auditory canal. The subcutaneous tissue was bluntly separated to expose the temporal bone, and the surface tissue was removed using 3% hydrogen peroxide. When the surface of the temporal bone was dry, the probe base was fixed on the smooth surface of the temporal bone using Loctite 4161 instant adhesive (Loctite, Hartford, CT, USA). rCBF was monitored by inserting the probe into the pedestal. After monitoring was completed, the skin was sutured using a 5-0 surgical suture, and iodine and penicillin sodium were used to disinfect the local skin. The rats were kept in cages after awakening.

### 2.5. Open-Field Test (OFT)

The OFT is widely used to assess anxiety-like behavior in rodents [30]. A square black box (100 cm wide and 50 cm high) with a camera above the center was used to perform this test. The bottom of the box was virtually divided into 16 squares (25 cm × 25 cm), and the four squares in the middle were termed the center area. The rats were gently placed in the center area at the start of the experiment. The activity of each rat in a 5 min period was then recorded and analyzed using an automatic image tracking system (SMART3.0, Panlab, Spanish). The parameters recorded included time spent in the center area, number of entries into the center area, and total distance traveled. After each rat was tested, the bottom and sides of the box were thoroughly scrubbed with 70% alcohol to remove feces and other odors, preventing these from affecting the behavior of other rats.

### 2.6. Novel-Object Recognition Test (NORT)

The NORT is a test of learning and memory based on the principle that animals innately explore new objects. It is a method commonly employed to assess hippocampal-dependent memory in experimental animals [31]. In the NORT, rats are allowed to perform learning and memory tests in a freely active state, which more closely simulates human learning and memory behavior. A square PVC plastic box with no lid (70 cm × 70 cm × 50 cm) and three objects were used to perform this test. The three objects comprised two identical cuboids, 8 cm in length, 8 cm in width, and 6 cm in height (object A), and a cylinder with a diameter of 7 cm and height of 10 cm (object B). The objects could not be moved by the rats. The experiment consisted of three phases, namely, adaptation, familiarity, and detection phases [32, 33]. The rats were stroked daily before starting the test to avoid stress responses during the test. In the adaptation phase, rats were gently placed in the box and allowed to move around freely for 10 min. In the familiar phase, two identical objects were placed in the same corner of the box, 10 cm away from the side. The rat was then gently placed next to the inner wall of the box away from the object and allowed to move freely for 5 min. The detection phase was carried out after 24 h. In the detection phase, one object A was replaced by the novel object B, and a camera recorded the exploration of the rat for 5 min. This test was initiated on the 20^th^ day after 2VO. A rat was considered to be exploring an object when its head was within 2 cm of the object. Object A was termed the familiar object, and object B was termed the novel object. Analysis was performed using SMART3.0 (Panlab, Spanish). The discrimination index (d1 = TN − TF) and the discrimination ratio [d2 = (TN − TF)/(TN + TF)] were calculated, where TN is the amount of time the rat spent exploring the novel object and TF is the amount of time the rat explored the familiar object. After each rat was tested, the box and objects were thoroughly scrubbed with 70% alcohol to remove feces and other odors, preventing these from affecting the behavior of other rats.

### 2.7. Morris Water Maze (MWM) Test

The MWM is a test of hippocampal-dependent spatial learning and memory in rodents [34]. The test was initiated on the 23^rd^ day after 2VO and lasted for 6 consecutive days. The first phase was a 5-day continuous navigation test which evaluated the learning ability of the rats. Rats need to find a platform (diameter, 10 cm) hidden 2 cm underwater within 1 min. After the rats climbed onto the platform, they needed to remain on the platform for at least 10 s. If rats did not find the hidden platform within 1 min, the experimenter would guide the rats to the platform and maintain it there for 30 s. The escape latency is the time taken for a rat to find the platform. Rats underwent four trials per day for 5 days and were excluded if they failed to find the platform on the 5^th^ day. The second phase, named the spatial probe test, on the 6^th^ day, was performed to test the memory ability of the rats by observing the time spent in the quadrant that previously contained the platform. The activity of the rats was recorded and analyzed using SMART3.0 software (Panlab, Spanish).

### 2.8. Enzyme-Linked Immunosorbent Assay (ELISA)

Rats were sacrificed after urethane anesthesia (1.2 g/kg i.p., Sigma-Aldrich). Animals were perfused with 4°C saline, and hippocampal tissues were quickly removed and placed in freeze storage at −70°C. Thawed hippocampal samples were homogenized in lysis buffer containing a protease inhibitor cocktail (Applygen, Beijing, China). The levels of ACE, ACE2, Ang I, Ang II, and Ang-(1-7) were measured in the supernatants using a Parameter™ ACE (Nanjing Jiancheng Bioengineering Institute, China), ACE2 (Anaspec, USA), Ang I (R&D Systems, USA), Ang II (R&D Systems, USA), or Ang-(1-7) (R&D Systems, USA) immunoassay kit, respectively, according to the manufacturer's instructions. The absorbance at 405 nm was read using a SpectraMax M4 microplate reader (Molecular Devices, USA), and the concentration was determined by fitting curves using SoftMax Pro software (version 5.0, Molecular Devices, USA). Five rats from each group were randomly selected for ELISA.

### 2.9. Western Blotting

The unfrozen hippocampal samples were homogenized with lysis buffer containing a cocktail of phosphatase and proteinase inhibitors and PMSF (Beyotime, Shanghai, China). After denaturation, the lysates were separated on a 10% SDS-PAGE gel and transferred to polyvinylidene difluoride (PVDF) membranes (Bio-Rad, USA). The membranes were blocked with 5% non-fat powdered milk in TBST (containing 0.1% Tween 20) for 1 h at 25°C and incubated for 15−18 h at 4°C with a monoclonal rabbit anti-MasR (1 : 2000, Proteintech Group), anti-AT1R (1 : 500, Proteintech Group), or anti-GAPDH (1 : 5000, Proteintech Group) primary antibody. After TBST cleaning, the membrane was incubated for 1 h at 25°C with a horseradish peroxidase- (HRP-) conjugated goat anti-rabbit antibody (1 : 5000, Jackson ImmunoResearch Laboratories, USA) and the protein bands were visualized using an ECL system (Immun-Star™ HRP Chemiluminescence Kit, Bio-Rad). The band images were recorded using the ImageQuant LAS 4000 system (GE Healthcare, Japan), and the band intensities were quantified using ImageQuant TL software (version 7.0, GE Healthcare, Japan). Five rats from each group were randomly selected for western blotting.

### 2.10. Immunofluorescence

After the sacrificed animals were perfused with 4°C saline and 4°C paraformaldehyde (PFA), the brains were removed and placed in 4°C paraformaldehyde for 24 h. After 15% and 30% sucrose gradient dehydration, the brain tissues were stored at -70°C until they completely sank to the bottom. A cryostat microtome (Microm HM 550, Thermo Fisher Scientific Inc., Germany) was used to cut 20 *μ*m thick coronal brain slices for immunofluorescence. Slices containing the hippocampal CA1, CA3, and DG region (−2.3 mm to −4.16 mm from the bregma) were chosen by the experimenters. Brain slices were blocked with 10% normal goat serum solution at 37°C for 2 h and then incubated for 18−20 h at 4°C with an anti-MasR antibody (rabbit polyclonal, 1 : 500, Alomone, Israel) or an anti-AT1R antibody (rabbit polyclonal, 1 : 500, Alomone, Israel). After washing with PBS, the slices were incubated with an Alexa Fluor 647-conjugated goat anti-rabbit secondary antibody (1 : 1000, Jackson ImmunoResearch Laboratories, USA) at 37°C for 1.5 h. Finally, the slices were visualized using a Nikon Eclipse Ti confocal microscope (Nikon, Japan) and images in the hippocampal CA1, CA3, and DG region were captured using NIS-Elements software (Nikon, Japan).

### 2.11. Statistical Analysis

All data were expressed as mean ± standard error (mean ± S.E.). Data from rCBF monitoring and the MWM positioning navigation test were analyzed using repeated measures analysis of variance (RM ANOVA). The remaining data were analyzed using a one-way ANOVA. If the variance was uniform, the Bonferroni test was used. If the variance was not uniform, Dunnett's T3 test was used. *P* < 0.05 was considered statistically significant.

## 3. Results

**Figure 1:**
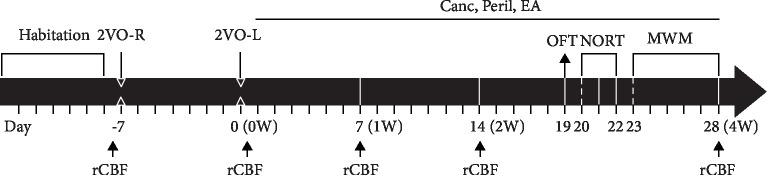
The basic experimental procedure. Abbreviations: Canc = candesartan cilexetil; Peril = perindopril; EA = electroacupuncture; 2VO-R = right common carotid artery occlusion; 2VO-L = left common carotid artery occlusion; OFT = open-field test; NORT = novel-object recognition test; MWM = Morris water maze; rCBF = regional cerebral blood flow; W = week.

As shown in Figure 1, after each group of SHR was acclimated for 1 week, the 2VO model induction was initiated. On day 7, the right common carotid artery (2VO-R) was occluded; 1 week later, the left common carotid artery (2VO-L) was occluded using the same method. Following this preparation of the CCH model, the EA, Canc, and Peril interventions were initiated for 28 days (4 weeks).

The rCBF in each group was measured before 2VO-R (baseline), immediately after 2VO-L, and 1, 2, and 4 weeks after 2VO-L. The OFT was performed on day 19, the NORT was performed on days 20−22, and the MWM test was performed on days 23−28. After the end of the MWM test on day 28, the rats were anesthetized and sacrificed, and ELISA, immunofluorescence, and immunoblotting were conducted.

### 3.1. EA Enhanced CCH-Induced Low rCBF in SHR

In this experiment, we used laser Doppler flowmetry to monitor rCBF in rats before 2VO-R, immediately after 2VO-L, and 1, 2, and 4 weeks after 2VO-L. Pre-2VO rCBF (baseline) showed no significant difference between the groups (*P* > 0.05, one-way ANOVA).

**Figure 2:**
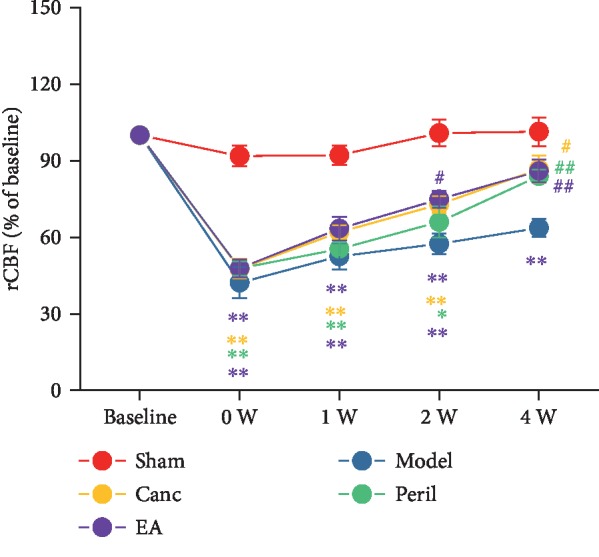
Changes in regional cerebral blood flow (rCBF) in each group before (baseline) and after two-vessel occlusion (2VO). Abbreviations: Canc = candesartan cilexetil; Peril = perindopril; EA = electroacupuncture; rCBF = regional cerebral blood flow; W = week. ^∗^*P* < 0.05 and ^∗∗^*P* < 0.01 compared to the sham group; ^#^*P* < 0.05 and ^##^*P* < 0.01 compared to the model group.

The results of the spherical test showed that rCBF was correlated with both the group and time after the intervention. Therefore, the data from this experiment were statistically processed using two-way repeated measures ANOVA. The results showed that compared to the sham group, the rCBF of the model group was significantly lower at each time point after 2VO (one-way RM ANOVA; all *P* < 0.01, Bonferroni test; Figure 2). Furthermore, compared to the sham group, the Canc, Peril, and EA groups exhibited significantly lower rCBF immediately after 2VO (one-way RM ANOVA; all *P* < 0.01, Bonferroni test; Figure 2), 1 week after 2VO (one-way RM ANOVA; all *P* < 0.01, Bonferroni test; Figure 2), and 2 weeks after 2VO (one-way RM ANOVA; *P* < 0.05 or *P* < 0.01, Bonferroni test; Figure 2). However, the Canc, Peril, and EA groups did not exhibit significantly different rCBF compared to that of the sham group 4 weeks after 2VO (one-way RM ANOVA; *P* > 0.05, Bonferroni test; Figure 2).

Compared to the model group, the rCBF of the Canc and Peril groups was not significantly different immediately after, 1 week, or 2 weeks after 2VO (one-way RM ANOVA; all *P* > 0.05, Bonferroni test; Figure 2). However, these groups exhibited significantly higher rCBF compared to the model group 4 weeks after 2VO (one-way RM ANOVA; *P* < 0.01, Bonferroni test; Figure 2). Compared to the model group, the rCBF of the EA group was not significantly different immediately or 1 week after 2VO (one-way RM ANOVA; both *P* > 0.05, Bonferroni test; Figure 2) but was significantly higher 2 and 4 weeks after 2VO (one-way RM ANOVA; *P* < 0.05 or *P* < 0.01, Bonferroni test; Figure 2). There were no significant differences in rCBF between the Canc, Peril, and EA groups at any of the recorded time points (one-way RM ANOVA; all *P* > 0.05, Bonferroni test; Figure 2).

### 3.2. EA Attenuated CCH-Induced Anxiety-Like Behavior in SHR

**Figure 3:**
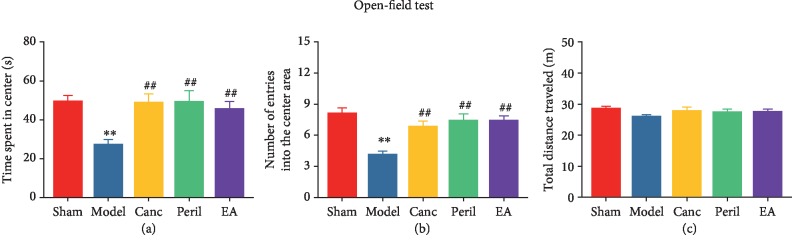
Changes in anxiety-like behavior in each group. Abbreviations: Canc = candesartan cilexetil; Peril = perindopril; EA = electroacupuncture. ^∗∗^*P* < 0.01 compared to the sham group; ^#^*P* < 0.05 and ^##^*P* < 0.01 compared to the model group.

The results of the OFT showed that compared to the sham group, the time spent in the central area (Figure 3(a)) and number of entries into the central area (Figure 3(b)) were significantly reduced in the model group (one-way ANOVA; all *P* < 0.01, Bonferroni test). Compared to the model group, the time spent in the central area (Figure 3(a)) and the number of entries into the central area (Figure 3(b)) were significantly lower in the Canc, Peril, and EA groups (one-way ANOVA; *P* < 0.05 or *P* < 0.01, Bonferroni test). There were no significant differences in the total distance traveled between five groups (*P* > 0.05, one-way ANOVA; Figure 3(c)).

### 3.3. EA Attenuated CCH-Induced Object Recognition Memory Impairments in SHR

**Figure 4:**
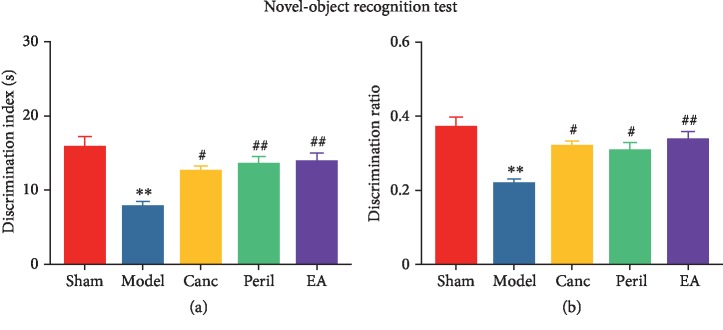
Changes in object recognition memory in each group. Abbreviations: Canc = candesartan cilexetil; Peril = perindopril; EA = electroacupuncture. ^∗∗^*P* < 0.01 compared to the sham group; ^#^*P* < 0.05 and ^##^*P* < 0.01 compared to the model group.

In the NORT, compared to the sham group, the discrimination index (d1, Figure 4(a)) and discrimination ratio (d2, Figure 4(b)) of the model group were significantly lower (one-way ANOVA; both *P* < 0.01, Bonferroni test; Figures 4(a) and 4(b)). Compared to the model group, the d1 and d2 of the Canc, Peril, and EA groups were significantly higher (one-way ANOVA; *P* < 0.05 or *P* < 0.01, Bonferroni test; Figure 4(a) and 4(b)). There were no significant differences in d1 or d2 between the Canc, Peril, and EA groups (one-way ANOVA; both *P* > 0.05, Bonferroni test).

### 3.4. EA Attenuated CCH-Induced Spatial Learning and Memory Impairments in SHR

**Figure 5:**
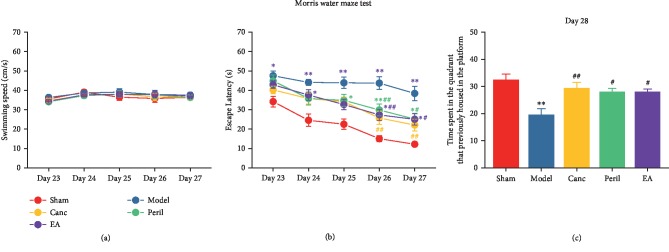
Changes in spatial learning (a, b) and memory (c) in each group. Abbreviations: Canc = candesartan cilexetil; Peril = perindopril; EA = electroacupuncture. ^∗^*P* < 0.05 and ^∗∗^*P* < 0.01 compared to the sham group; ^#^*P* < 0.05 and ^##^*P* < 0.01 compared to the model group.

The spatial learning performance of the rats was observed during 5 days of MWM training. There was no significant difference in the swimming speed of the SHR treated with different interventions (*P* > 0.05, two-way RM ANOVA; Figure 5(a)). This eliminated the possible impact of the 2VO model itself on the free movement of rats. Compared to the sham group, the escape latency of the model (days 23-27), Peril (days 25-27), and EA (day 24, days 26-27) groups was significantly higher (one-way RM ANOVA; *P* < 0.05 or *P* < 0.01, Bonferroni test; Figure 5(b)). Compared to the model group, the escape latency of the Canc (days 26-27), Peril (days 26-27), and EA (days 26-27) groups was significantly lower (one-way RM ANOVA; *P* < 0.05 or *P* < 0.01, Bonferroni test; Figure 5(b)).

After 5 days of training, on day 28, the platform was removed to test the spatial memory performance of the rats. Relative to the sham group, the time spent in the quadrant that previously contained the platform was significantly decreased in the model group (one-way ANOVA; *P* < 0.01, Bonferroni test; Figure 5(c)). Relative to the model group, the time spent in the quadrant that previously contained the platform was significantly increased in the Canc, Peril, and EA groups (one-way ANOVA; *P* < 0.05 or *P* < 0.01, Bonferroni test; Figure 5(c)).

### 3.5. EA Attenuated CCH-Induced Increased Hippocampal ACE Activity, ACE2 Activity, and Ang I Level in SHR

**Figure 6:**
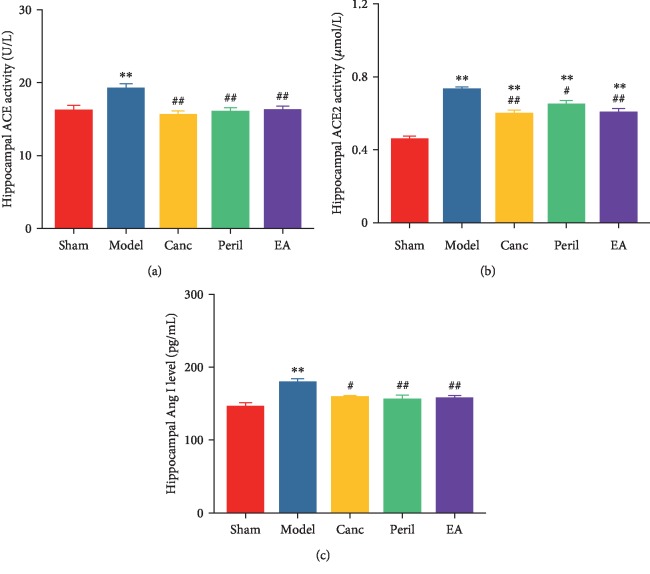
Changes in hippocampal ACE activity (a), ACE2 activity (b), and Ang I level (c) in each group. Abbreviations: ACE = angiotensin converting enzyme; ACE2 = angiotensin I converting enzyme 2; Ang I = angiotensin I; Canc = candesartan cilexetil; Peril = perindopril; EA = electroacupuncture. ^∗∗^*P* < 0.01 compared to the sham group; ^#^*P* < 0.05 and ^##^*P* < 0.01 compared to the model group.

Relative to the sham group, hippocampal ACE activity was significantly higher in the model group (one-way ANOVA; *P* < 0.01, Bonferroni test; Figure 6(a)). Relative to the model group, hippocampal ACE activity was significantly lower in the Canc, Peril, and EA groups (one-way ANOVA; all *P* < 0.01, Bonferroni test; Figure 6(a)). There was no significant difference in ACE activity between the Canc, Peril, and EA groups (one-way ANOVA; *P* > 0.05, Bonferroni test).

Relative to the sham group, hippocampal ACE2 activity was significantly higher in the model, Canc, Peril, and EA groups (one-way ANOVA; all *P* < 0.01, Bonferroni test; Figure 6(b)). Relative to the model group, hippocampal ACE2 activity was significantly lower in the Canc, Peril, and EA groups (one-way ANOVA; *P* < 0.05 or *P* < 0.01, Bonferroni test; Figure 6(b)). There was no significant difference in hippocampal ACE2 activity between the Canc, Peril, and EA groups (one-way ANOVA; *P* > 0.05, Bonferroni test).

Relative to the sham group, the hippocampal Ang I level was significantly higher in the model group (one-way ANOVA; *P* < 0.01, Bonferroni test; Figure 6(c)). Relative to the model group, the hippocampal Ang I level was significantly lower in the Canc, Peril, and EA groups (one-way ANOVA; *P* < 0.05 or *P* < 0.01, Bonferroni test; Figure 6(c)). There was no significant difference in the hippocampal Ang I level between the Canc, Peril, and EA groups (one-way ANOVA; *P* > 0.05, Bonferroni test).

### 3.6. EA Attenuated CCH-Induced Increased Hippocampal Ang II Level and Ang II/Ang-(1-7) Ratio in SHR

**Figure 7:**
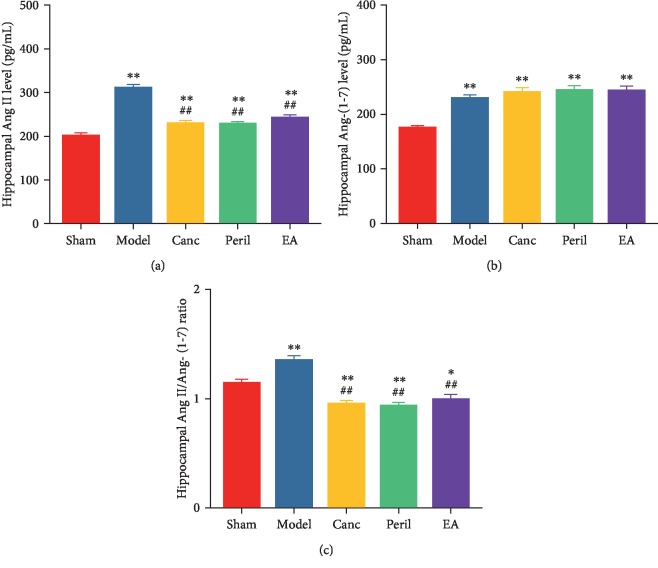
Changes in hippocampal Ang II level (a), Ang-(1-7) level (b), and Ang II/Ang-(1-7) ratio (c) in each group. Abbreviations: Ang II = angiotensin II; Ang-(1-7) = angiotensin 1-7; Canc = candesartan cilexetil; Peril = perindopril; EA = electroacupuncture. ^∗^*P* < 0.05 and ^∗∗^*P* < 0.01 compared to the sham group; ^##^*P* < 0.01 compared to the model group.

Relative to the sham group, the hippocampal Ang II level was significantly higher in the model, Canc, Peril, and EA groups (one-way ANOVA; all *P* < 0.01, Bonferroni test; Figure 7(a)). Relative to the model group, the hippocampal Ang II level was significantly lower in the Canc, Peril, and EA groups (one-way ANOVA; all *P* < 0.01, Bonferroni test; Figure 7(a)). There was no significant difference in the hippocampal Ang II level between the Canc, Peril, and EA groups (one-way ANOVA; all *P* > 0.05, Bonferroni test).

Relative to the sham group, the Ang-(1-7) level was significantly higher in the model, Canc, Peril, and EA groups (one-way ANOVA; all *P* < 0.01, Bonferroni test; Figure 7(b)). There was no significant difference in the hippocampal Ang-(1-7) level between the Canc, Peril, and EA groups (one-way ANOVA; all *P* > 0.05, Bonferroni test).

Furthermore, relative to the sham group, the Ang II/Ang-(1-7) ratio was significantly higher in the model group (one-way ANOVA; *P* < 0.01, Bonferroni test; Figure 7(c)) and significantly lower in the Canc, Peril, and EA groups (one-way ANOVA; *P* < 0.05 or *P* < 0.01, Bonferroni test; Figure 7(c)). Relative to the model group, the Ang II/Ang-(1-7) ratio was significantly lower in the Canc, Peril, and EA groups (one-way ANOVA; all *P* < 0.01, Bonferroni test; Figure 7(c)). There was no significant difference in the hippocampal Ang II/Ang-(1-7) ratio between the Canc, Peril, and EA groups (one-way ANOVA; all *P* > 0.05, Bonferroni test).

### 3.7. EA Attenuated CCH-Induced Increased Hippocampal AT1R Expression and Strengthened CCH-Induced Reduced Hippocampal MasR Expression in SHR

**Figure 8:**
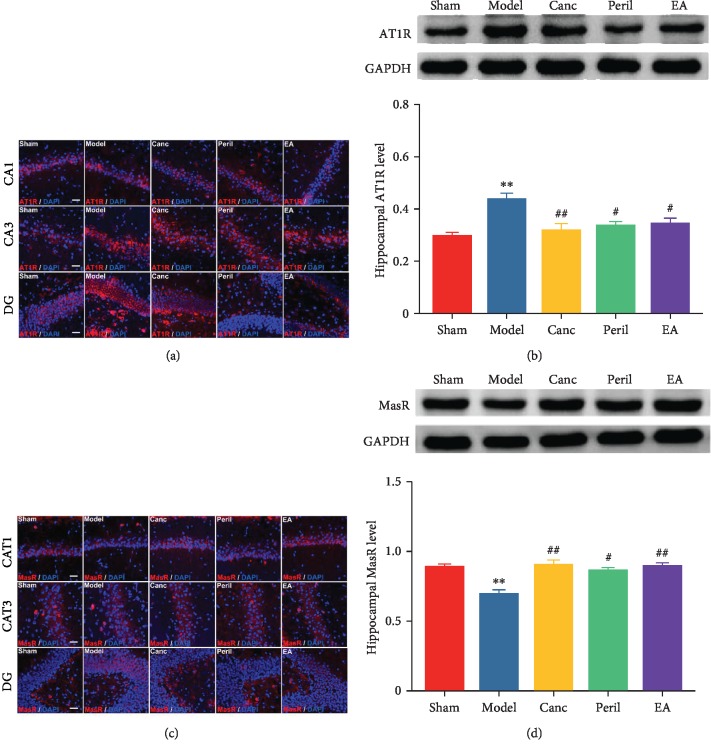
Changes in hippocampal AT1R (a, b) and MasR levels (c, d) in each group. Abbreviations: AT1R = angiotensin II type 1 receptor; MasR = Mas receptor; Canc = candesartan cilexetil; Peril = perindopril; EA = electroacupuncture. ^∗∗^*P* < 0.01 compared to the sham group; ^#^*P* < 0.05 and ^##^*P* < 0.01 compared to the model group.

The results of AT1R immunofluorescence (Figure 8(a)) showed that AT1R (red) was expressed in the CA1, CA3, and DG area of the hippocampus, and AT1R-positive cells were costained with the nuclear stain DAPI (blue).

When AT1R expression was quantified relative to that of GAPDH (internal reference protein) (Figure 8(b)), relative to the sham group, the expression of AT1R was significantly higher in the model group (one-way ANOVA; *P* < 0.01, Bonferroni test; Figure 8(b)). Relative to the model group, the expression of AT1R was significantly lower in the Canc, Peril, and EA groups (one-way ANOVA; *P* < 0.05 or *P* < 0.01, Bonferroni test; Figure 8(b)). There was no significant difference in AT1R expression between the Canc, Peril, and EA groups (one-way ANOVA; all *P* > 0.05, Bonferroni test).

The results of MasR immunofluorescence (Figure 8(c)) showed that MasR (red) was expressed in the CA1, CA3, and DG area of the hippocampus and MasR-positive cells were costained with the nuclear stain DAPI (blue).

The expression of MasR was quantified relative to that of GAPDH using western blotting (Figure 8(d)). Relative to the sham group, MasR protein expression was significantly lower in the model group (one-way ANOVA; *P* < 0.01, Bonferroni test; Figure 8(d)). Relative to the model group, the expression of MasR was significantly higher in the Canc, Peril, and EA groups (one-way ANOVA; *P* < 0.05 or *P* < 0.01, Bonferroni test; Figure 8(d)). There was no significant difference in MasR expression between the Canc, Peril, and EA groups (one-way ANOVA; all *P* > 0.05, Bonferroni test).

## 4. Discussion

In this study, the OFT, NORT, and MWM test were used to observe the effects of CCH on anxiety, recognition memory, and spatial learning and memory in SHR and to investigate how these were affected by EA. Previous studies have shown that CCH can cause anxiety [35] and memory impairment [36], but it is still unclear whether CCH combined with hypertension also has similar detrimental effects. Our results show that CCH induces memory impairments and anxiety in SHR and EA can effectively alleviate CCH-induced anxiety and memory impairment in SHR.

Accumulating evidence has proven that angiotensinogen (AGT) is the sole precursor protein that harbors Ang I at the N-terminus, which is specifically cleaved by renin [37]. Ang II is obtained by a strong vasoconstrictor (ACE) cleaving Ang I [38]. The combination of Ang II and AT1R could induce vascular remodeling, promote vascular inflammation and oxidative stress injury, and subsequently cause brain damage [39, 40]. The accepted view is that Ang II in the brain acts through a complex neural network to mediate hypertension [41, 42]. Ang II infusion in chronic hypertension induces learning and spatial memory impairment, as well as anxiety [43]. Furthermore, the activation of the ACE/Ang II/AT1R axis also causes no hypertensive disorders, such as cerebral hypoperfusion and ischemic injury [44–46]. Those disorders induced anxiety-like behavior and memory loss [14, 47]. In our study, relative to SHR, CCH in SHR induced significantly activation of the ACE/Ang II/AT1R axis and exacerbated anxiety-like behavior and memory deficits. These indicate that the ACE/Ang II/AT1R axis is involved in the superposition effect of CCH and causes more serious functional damage.

In addition to binding to AT1R, a part of Ang II is hydrolyzed to Ang-(1-7) by ACE2, which plays a neuroprotective role by acting on the MasR, exerting anxiolytic effects [48] and ameliorating memory impairment [49]. Studies have shown that hypertension could induce decreased ACE2 levels and reduced generation of Ang-(1-7) [50]. Overexpression of ACE2 could decrease hypertension in SHR [51]. Emerging evidence suggests that the activation of the ACE2-Ang-(1-7)-MasR axis could attenuate the development of hypertension and pathologic progress of atherosclerosis [52]. However, in ischemic cerebrovascular diseases, ischemic stroke could induce the upregulation of ACE2, Ang-(1-7), and MasR in the brain [53, 54]. In our study, relative to SHR, CCH in SHR induced activation of hippocampal ACE2 and Ang-(1-7), but the Ang II level was much higher than that of Ang-(1–7), i.e., the Ang II/Ang-(1-7) ratio remained high. Those indicate that the CCH-induced increase of ACE2 and Ang-(1-7) could result in a partial and limited self-protective response and does not relieve memory impairment and anxiety-like behavior in SHR.

In this study, Canc and Peril were used as positive control drugs. Canc is an AT1R antagonist typically used for the treatment of hypertension. Studies showed that Canc inhibits the actions of Ang II by specifically binding with AT1R in the central nervous system, reducing oxidative stress, increasing cerebral blood flow, and protecting against CCH-induced memory damage [1, 55–57]. It is also documented that the anxiolytic effect of the Canc can be observed in the elevated plus maze [58]. Furthermore, Peril, a classical ACE inhibitor, is used to treat hypertension by blocking the conversion of Ang I to Ang II and decreasing the expression of Ang II. Peril could be useful for preventing CCH-induced complications, such as memory deficits and dysphagia [59, 60], but the effect of Peril on CCH-induced anxiety-like behavior is not clear.

In our studies, EA and the two positive control drugs (Canc and Peril) generated similar relieving effects on CCH-induced memory loss and anxiety in SHR. After EA, ACE activity, Ang I level, Ang II level, AT1R expression, and the ratio of Ang II to Ang-(1-7) all decreased, and ACE2 activity, Ang-(1-7) level, and MasR expression all increased. We speculate that although EA is not as targeted as the two positive control drugs, the effect of EA may be multitargeted. EA may make the ACE/Ang II/AT1R axis and ACE2/Ang-(1-7)/MasR axis reach a new balance by downregulating the ACE/Ang II/AT1R pressor axis and upregulating the ACE2/Ang-(1-7)/MasR protective axis.

## 5. Conclusions

In summary, EA can improve CCH-induced anxiety-like behavior and memory impairments in SHR. EA therapy for CCH with hypertension may be mediated via downregulation of the hippocampal ACE/Ang II/AT1R axis and upregulation of the hippocampal ACE2/Ang-(1-7)/MasR axis.

## Data Availability

The data used to support the findings of this study are available from the corresponding author upon request.
